# “Fixing a heart”: the game of electrolytes in anorexia nervosa

**DOI:** 10.1186/1475-2891-13-90

**Published:** 2014-09-05

**Authors:** Jean Abed, Hani Judeh, Elie Abed, Matthew Kim, Haword Arabelo, Rajan Gurunathan

**Affiliations:** Department of Medicine, Icahn School of Medicine, Mount Sinai St. Luke’s and Mount Sinai Roosevelt Hospitals, New York, NY USA; Medical Student, College of Physician and Surgeon, Columbia University, New York, NY USA

## Abstract

**Case:**

A 25-year-old woman with chronic anorexia nervosa and depression presented with sudden weakness and fatigue. Psychosocial history was notable for binge-starve cycles over the past year and a decline in overall well-being. Vitals on presentation were notable for hypothermia, hypotension, and bradycardia. Initial exam was significant for emaciation, lethargy, and lower extremity edema. Laboratory work-up revealed markedly elevated LFTs, hypoglycemia, thrombocytopenia and elevated INR and lipase. ECG showed sinus bradycardia with prolonged QTc. Ultrasound revealed normal liver and biliary tree. Serum acetaminophen, alcohol level, and urinary toxicology were unremarkable. Work up for infectious, autoimmune, and genetic causes of hepatitis was negative. Echocardiogram revealed left ventricular hypokinesis and EF 10-15%. Nutritional support was begun slowly, however electrolyte derangements began to manifest on hospital day 2, with hypophosphatemia, hypokalemia, hypocalcemia, and hypomagnesemia. Multiple medical and psychiatric disciplines were consulted, and aggressive electrolyte monitoring and repletion were done. The patient’s overall clinical status improved slowly during her hospital course. Her liver enzymes trended down, and her QTc interval eventually returned toward the normal range. Repeat echocardiogram following treatment revealed improvement of her EF to 40%.

**Discussion:**

Anorexia nervosa is an eating disorder characterized by extremely low body weight, fear of gaining weight or distorted perception of body image, and amenorrhea. Anorexia can lead to life threatening medical complications, and thus constitutes a major challenge to manage. Central to the pathogenesis of the refeeding syndrome is a weakened cardiopulmonary system, electrolytes abnormalities, hepatic dysfunction, liver hypoperfusion and failure.

**Conclusion:**

Given the clinical presentation, this patient likely presented on the brink of developing frank refeeding syndrome, with cardiac dysfunction and hypovolemia, leading to hepatic hypoperfusion and ischemic hepatitis. Subsequently, she developed electrolyte disturbances characteristic of refeeding syndrome, which were managed without major complication. Her hospital course is encouraging not only for her recovery, but for the collaboration of the different teams involved in her care, and it highlights the importance of a multidisciplinary approach to caring for patients with the potential dire complications of a complex psychiatric illness.

## Case

A 25 year-old woman with a past medical history significant for anorexia nervosa, for which she had multiple psychiatric admissions, depression and Attention deficit hyperactivity disorder on fluoxetine and methylphenidate who presented with weakness for 1 day. She was in her usual state of health until the night prior to presentation, when she began to feel weak after having dinner. The next morning she had difficulty getting out of bed, felt dehydrated and was lethargic. The patient reports that her “normal” weight is between 90 and 100 pounds. Recently, she moved out of her parents’ home, withdrew socially, resumed binging, and started to feel depressed. Since then, her eating habits have been characterized by cycles of binging and starving, during which she will consume an entire box of pasta or 20 apples, feel sick, and then starve until feeling hungry again. She denies fever, nausea and vomiting, diarrhea, urinary symptoms, and recent increase in exercise volume or frequency. She also denies current suicidal and homicidal ideation.

In the emergency room, vital signs were significant for rectal temperature 93 F, heart rate 56BPM, blood pressure 80/55, and respiratory rate 14. Physical exam was remarkable for lethargy, emaciation and pitting lower extremity edema. Electrocardiogram showed sinus bradycardia, with a prolonged corrected QT interval of 0.54 and U waves, attributed initially to magnesium deficiency. She was given 2 grams of magnesium intravenously in the emergency department. Laboratory studies (Table 1) were notable for BUN 37 and Cr 0.6, glucose 31, Ca 7.9, Mg 1.9, phos 3.5, AST 1,386, ALT 1,208, alkaline phosphatase 378 and prolonged prothrombin time. She received 10% dextrose in water solution and was admitted to Medicine for further management of EKG abnormalities and elevated liver transaminases.

**Table 1:** **Important laboratory results from admission to discharge**

	On Admission	Day 2	On discharge (day 10)
Sodium (137–145 mmol/l)	133	130	132
Potassium (3.5-5.1 mmol/l)	2.7	3.4	3.6
Phosphorus (2.5-4.5 mg/dl)	3.5	2.1	3.4
Urea Nitrogen (7–17 mg/dl)	37	13	15
Creatinine (0.52-1.04 mg/dl)	0.6	0.3	0.3
Calcium (8.4-10.3 mg/dl)	7.9	6.7	7.8
Glucose (74–106 mg/dl)	31	73	65
AST (15–46 u/l)	1386	2833	537
ALT (13–69 u/l)	1208	2114	1050
Alkaline Phosphatase (38–126 u/l)	378	471	47
PT (11.8-14.5 sec)	22	23.1	15.3
INR (0/9-1.1)	1.9	2.1	1.2
PTT (25–36.6 sec)	36.1	26.6	34.4
Magnesium (1.6-2.3 mg/dl)	1.9	1.6	1.8
Hemoglobin (12–16 g/dl)	13.5	7.7	7.4
WBC count (4.5-10.8 K/ul)	3.1	1	1.9
Platelets (150–450 K/ul)	83	21	67

On the medical ward, patient was hypophosphatemic, hypokalemic, hypocalcemic, and hypomagnesemic, requiring IV fluids and frequent electrolytes repletion. She was closely monitored with multiple daily blood draws. Working closely with the Nutrition and Endocrine services, Her diet was slowly advanced with increase in calorie allowance. Throughout her nutritional rehabilitation, she had a vigorous appetite and had no problems eating or voiding.

**Figure 1:**
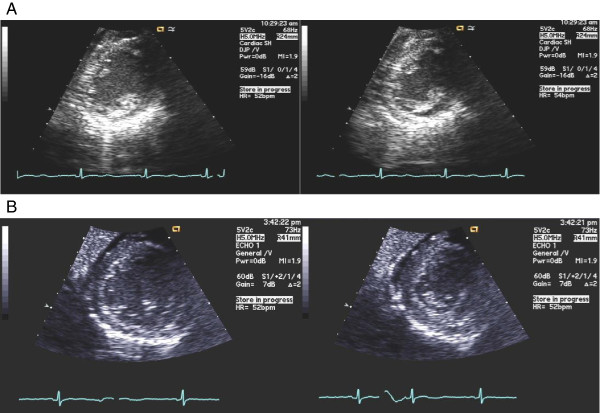
**TransThoracic Echocardiogram (TTE). A**. Initial TTE: Left ventricular hypokinesis and EF 10-15%. **B**. Post recovery TTE: EF 40%.

Ultrasound of the right upper quadrant revealed a normal size liver, with no structural lesions, and a gallbladder polyp, with no evidence of cholestasis or cholelithiasis. Blood acetaminophen and alcohol levels and urinary toxicology were unremarkable. Work up for infectious, autoimmune, and genetic causes of hepatitis was unremarkable. During this time, the patient’s AST and ALT peaked in the high 4,000 s and slowly trended downward. She refused a liver biopsy. Ultimately, the patient’s liver failure was attributed to chronic malnutrition and possible liver hypoperfusion in the setting of poor oral intake.The patient remained in sinus rhythm on telemetry, with heart rate ranging from 40 and 100 beats per minute and persistent QTc prolongation. Trans-thoracic echocardiogram (Figure 1) revealed a hypokinetic left ventricle, left ventricular wall atrophy, and an ejection fraction of 10-15%. Following carddiology recommendations, the patient’s phosphorus, magnesium, and potassium were aggressively maintained at >3.5, >2.0, and >4.0, respectively, to reduce the risk of torsades de pointes. The corrected QT interval eventually downtrended toward the normal range.

The psychiatrist continued to meet and support the patient throughout her stay in the hospital and was present on rounds to discuss the patient’s discharge disposition and plans for follow up. Her hospital course was otherwise notable for steadily decreasing blood counts. During her hospitalization, she was found to be pancytopenic with an absolute neutrophil count as low as 700 and platelet count of 21,000. She was placed briefly on reverse contact isolation for infection prophylaxis until her white count and ANC increased.

The patient improved dramatically over the first few days and by day 4 of hospitalization, she was able to ambulate without assistance and use the bathroom independently she was discharged by day 10.

## Discussion

Anorexia nervosa is an eating disorder characterized by extremely low body weight, fear of gaining weight or distorted perception of body image, and amenorrhea [1]. Although described mostly in the context of psychiatric illness, anorexia can lead to devastating, and at times life threatening, medical complications and thus constitutes a challenging condition to manage [2]. Serious complications include electrolyte disturbances, often in the context of the refeeding syndrome, hypothermia, endocrine dysfunction, and multi-organ failure. Anorexia nervosa is most likely responsible for our patient’s triad of weakness, liver dysfunction, and prolonged QT interval.

Refeeding malnourished patients with anorexia nervosa can be associated with hypophosphatemia, cardiac arrhythmia and delirium. Phosphorus repletion should be started early with and serum levels maintained above 3 mg/dL. Patients need close monitoring since cardiac and neurologic events associated are most likely to occur within the first weeks [3]. In chronically malnourished anorexia nervosa patients, a slow and a gradual increase in nutrition with nutritional counseling, psychotherapy and careful monitoring of body weight, heart rate and rhythm and serum electrolytes is recommended to deliver a safe and effective nutritional rehabilitation and to avoid rapid electrolyte shifts and fluid overload [4–6].

The patient’s weakness and fatigue, in the context of a recent history of starting a regular diet while in a state of chronic malnutrition, are concerning for refeeding syndrome, which typically occurs 2 to 5 days after beginning nutritional repletion [7]. Depleted phosphate stores due to prolonged starvation, hypocalcemia, and hypokalemia can lead to impaired muscle contractility and subsequently weakness, myalgia, and tetany. Hypoglycemia and anemia or pancytopenia from chronic malnutrition may have also contributed to the patient’s weakness.

Liver injury with an elevation of liver enzymes is a frequent complication, and steatosis of the liver is thought to be the major underlying pathology. The treatment is hydration, correction of electrolytes and fluid imbalance, and gradual nutritional support to prevent refeeding syndrome [8]. The patient exhibits elevated serum transaminases, elevated alkaline phosphatase, and prolonged prothrombin time, which collectively are suggestive of acute liver failure. Liver dysfunction likely also contributed to the patient’s hypoglycemia by compromising hepatic gluconeogenesis. Liver hypoperfusion due to anorectic hypovolemia, phosphate and thiamine depletion secondary to refeeding syndrome may have synergistically caused rapid and profound injury to the hepatocytes, resulting in the leak of alanine and aspartate aminotransferases into the serum.

Bradycardia is a common finding in patients with anorexia nervosa secondary to hypothermia and perhaps as a compensatory mechanism to conserve energy in states of starvation. Hypokalemia, hypomagnesemia, and hypocalcemia are typical findings in the refeeding syndrome and may be contributing to the patient’s EKG abnormalities showing prolonged QTc [9].

Electrolyte abnormalities occur in anorexia nervosa most often in the context of the refeeding syndrome, defined in 1990 by Solomon and Kirby as “the metabolic and physiologic consequences of the depletion, repletion, compartmental shifts and interrelationships of phosphorus, potassium, magnesium, glucose metabolism, vitamin deficiency and fluid resuscitation” [10]. Central to the pathogenesis of the refeeding syndrome is a weakened cardiopulmonary system, which is incapable of accommodating the fluid and sodium load presented to the body during nutritional repletion. The resultant volume expansion and fluid retention can progress even to heart failure. Typical electrolyte abnormalities include hypophosphatemia, hypomagnesemia, hypokalemia, and hypocalcemia, with deficiencies in thiamine and other B complex vitamins, as seen during this patient’s hospital course.

Hepatic dysfunction is a common medical complication of anorexia nervosa and its treatment. Its exact presentation and laboratory profile, however, remain widely variable, perhaps reflecting the lack of knowledge regarding the etiology and pathophysiology of this condition. One retrospective study of 126 patients with anorexia nervosa—with no history of prior liver disease, hepatotoxic drug exposure, or alcohol consumption—who were malnourished and subsequently hospitalized for parenteral nutrition found that 43% had elevated serum transaminases on admission [11]. The authors identified 4 risk factors associated with these laboratory findings: young age, low BMI, restrictive subtype of anorexia nervosa, and male gender. They also noted resolution of the transaminitis in most cases following nutritional repletion over a period of 4 weeks. Notably, the highest values observed for AST and ALT were 2,120 and 2,614, respectively, well below this patient’s peak values in the 4,000 s.

A case report from Japan in 1999 describes a 20 year-old woman with anorexia nervosa (BMI 12.1) who presented to the hospital with lethargy and lightheadedness and was found to have prolonged PT, thrombocytopenia to 64,000, and AST and ALT of 5,000 and 3,980, respectively [12]. She was treated with plasmapheresis for liver dysfunction, but subsequently developed pulmonary edema, acute renal failure, gastrointestinal bleeding, and disseminated intravascular coagulation. Extensive work up for infectious and drug-induced hepatitis was unremarkable, as was investigation for fatty liver changes and antioxidant deficiency. The authors stopped short of undertaking a liver biopsy, as the patient eventually recovered, but they concluded, by exclusion, that malnutrition itself might have led to hepatic failure.

More recently, a case report from the UK of a patient with anorexia nervosa and a BMI of 9 admitted for seizure also described elevation of AST to 5,403 and coagulopathy with an INR of 2.0 [13]. There was no history of alcohol or hepatotoxic drug use, and work up for viral and autoimmune hepatitis and paracetamol toxicity was unremarkable. The liver enzymes normalized spontaneously as the patient was nutritionally rehabilitated, and her acute liver failure was attributed to an episode of hypotension (BP 80/50). Hypotension was due to poor nutritional intake and secondary to cardiac dysfunction from chronic malnutrition led to liver hypoperfusion and, ultimately, ischemic hepatitis.

Further investigation of the patient’s cardiac abnormalities revealed left ventricular hypokinesis and EF 10-15%. Takotsubo cardiomyopathy, also known as stress-induced cardiomyopathy, has been described as a rare complication in young women with anorexia nervosa and usually presents in a manner similar to acute myocardial infarction [14]. The condition is characterized by transient hypokinesis, akinesis, or dyskinesis of the left ventricle with or without apical involvement; regional wall motion abnormalities extending beyond a single vascular distribution; presence of a stressful trigger in most cases; absence of coronary artery disease or evidence of plaque rupture; new EKG abnormalities or cardiac enzyme leak; and absence of pheochromocytoma and myocarditis. The pathophysiology of takotsubo cardiomyopathy remains to be elucidated but is thought to involve catecholamine excess leading to myocardial stunning.

Given the findings on history and physical exam and a review of relevant literature, the patient likely presented on the brink of developing frank refeeding syndrome with cardiac dysfunction and hypovolemia leading to hepatic hypoperfusion and ischemic hepatitis. Subsequently, she developed electrolyte disturbances characteristic of refeeding syndrome, which were managed without major complication. Her hospital course is encouraging not only for her recovery, but for the collaboration of the different teams involved in her care, and it highlights the importance of a multidisciplinary approach to caring for patients with the potential dire complications of a complex psychiatric illness.

## Consent

An oral over the phone consent was obtained from the patient for publication of this case report and any accompanying images.
